# Evidence of Improved Vascular Function in the Arteries of Trained but Not Untrained Limbs After Isolated Knee-Extension Training

**DOI:** 10.3389/fphys.2019.00727

**Published:** 2019-06-12

**Authors:** Angela Valentina Bisconti, Emiliano Cè, Stefano Longo, Massimo Venturelli, Giuseppe Coratella, Sheida Shokohyar, Reza Ghahremani, Susanna Rampichini, Eloisa Limonta, Fabio Esposito

**Affiliations:** ^1^Department of Biomedical Science for Health, Università degli Studi di Milano, Milan, Italy; ^2^Department of Internal Medicine, The University of Utah, Salt Lake City, UT, United States; ^3^Geriatric Research, Education, and Clinical Centre, Veterans Affairs Medical Centre, Salt Lake City, UT, United States; ^4^IRCCS, Istituto Ortopedico Galeazzi, Milan, Italy; ^5^Department of Neurological, Neuropsychological, Morphological and Movement Sciences, University of Verona, Verona, Italy; ^6^Department of Exercise Physiology, Faculty of Sport Sciences, University of Guilan, Rasht, Iran

**Keywords:** flow mediated dilation, single passive limb movement, shear rate, isolated leg extension muscle, reactive hyperaemia, vascular conditioning

## Abstract

Vascular endothelial function is a strong marker of cardiovascular health and it refers to the ability of the body to maintain the homeostasis of vascular tone. The endothelial cells react to mechanical and chemical stimuli modulating the smooth muscle cells relaxation. The extent of the induced vasodilation depends on the magnitude of the stimulus. During exercise, the peripheral circulation is mostly controlled by the endothelial cells response that increases the peripheral blood flow in body districts involved but also not involved with exercise. However, whether vascular adaptations occur also in the brachial artery as a result of isolated leg extension muscles (KE) training is still an open question. Repetitive changes in blood flow occurring during exercise may act as vascular training for vessels supplying the active muscle bed as well as for the vessels of body districts not directly involved with exercise. This study sought to evaluate whether small muscle mass (KE) training would induce improvements in endothelial function not only in the vasculature of the lower limb (measured at the femoral artery level in the limb directly involved with training), but also in the upper limb (measured at the brachial artery level in the limb not directly involved with training) as an effect of repetitive increments in the peripheral blood flow during training sessions. Ten young healthy participants (five females, and five males; age: 23 ± 3 years; stature: 1.70 ± 0.11 m; body mass: 66 ± 11 kg; BMI: 23 ± 1 kg ⋅ m^-2^) underwent an 8-week KE training study. Maximum work rate (MWR), vascular function and peripheral blood flow were assessed pre- and post-KE training by KE ergometer, flow mediated dilatation (FMD) in the brachial artery (non-trained limb), and by passive limb movement (PLM) in femoral artery (trained limb), respectively. After 8 weeks of KE training, MWR and PLM increased by 44% (*p* = 0.015) and 153% (*p* = 0.003), respectively. Despite acute increase in brachial artery blood flow during exercise occurred (+25%; *p* < 0.001), endothelial function did not change after training. Eight weeks of KE training improved endothelial cells response only in the lower limb (measured at the femoral artery level) directly involved with training, likely without affecting the endothelial response of the upper limb (measured at the brachial artery level) not involved with training.

## Introduction

Tobacco smoking, alcohol abuse, unbalanced diet, and physical inactivity, represent the main unhealthy habits, and their prevention, with an appropriate intervention, could reduce the number of premature deaths (World Health Organization [WHO], 2016). In particular, the introduction of regular physical activity is associated with numerous benefits on the overall cardiovascular risk, such as a reduction in arterial pressure, lipids level, insulin resistance, and exercise intolerance (Green et al., 2008, 2017a,b; Riebe et al., 2015; Adamson et al., 2018; Tomschi et al., 2018). However, since the modification of these risk factors taken alone underestimate the magnitude of exercise-induced risk reduction (Mora et al., 2007), it was recently proposed the so-called “vascular conditioning” theory to explain such a gap (Green et al., 2008, 2017a). This theory is based on the possible exercise-induced effects on vasculature structure and function (i.e., vasomotor response). The mechanical effects, provided by the repetitive increases in arterial pressure, blood flow (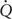), and shear rate (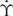) occurring on inner vessels wall during each training session (Green et al., 2017a), are the main stimuli able to enhance the endothelial cells response via an increase in several vasoactive factors bioavailability, among which the most common is NO (Joyner and Green, 2009; Naylor et al., 2011).

Exercise training protocols, generally, lead to a better vascular endothelial response mainly due to a shear-dependent mechanism (Tinken et al., 2009; Birk et al., 2012a,b; Green et al., 2017a). Briefly, 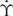 is the laminar shear force running in parallel to vessels’ long axis (Niebauer and Cooke, 1996). During exercise, 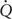 augmentation drives the increase in 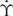, which stimulates endothelial cells to release vasoactive factors. However, the amount of skeletal muscles involved in the exercise could generate different 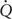 and 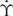 pattern according to the increase in muscles metabolic demands (Green et al., 2005; Thijssen et al., 2009a,b; Tinken et al., 2009; Spence et al., 2013). Indeed, it seems that repeated exercise sessions could have positive and beneficial effect on the overall endothelium health as a consequence of training-related changes in 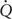 and 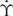 pattern (Spence et al., 2013; Kazmi et al., 2015; Davies, 2016; Green et al., 2017a,b).

Isolated knee extension muscles (KE) training is a type of small muscle mass exercise utilized in previous investigations (Wray et al., 2006; Esposito et al., 2010, 2011, 2018). Compared to other forms of exercise, KE exercise limits muscular work to the quadriceps muscle (Andersen et al., 1985). This exercise modality has been already used to train patients with chronic heart failure (Esposito et al., 2010, 2011, 2018) or with other pathologies characterized by a central limitation to aerobic exercise performance (Richardson et al., 2004). At the end of training, improvements in muscle structure, peripheral convective and diffusive oxygen transport, and subsequently, oxygen uptake (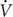
O_2_) were found (Esposito et al., 2010, 2011), thus supporting the efficacy of this small muscle mass training modality. Moreover, the 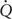 increment in the femoral artery during a single KE session (Paterson et al., 2005) is likely happening throughout all the training period, possibly triggering the cascade of events leading to a “vascular conditioning” in the artery directly involved in the exercise.

Improvements in endothelial cells function in arteries not directly involved in exercise (e.g., brachial artery) have been observed after whole body exercise training (i.e., cycling, running) (Birk et al., 2012a; Spence et al., 2013). Interestingly, positive vascular effects were also found after small mass muscle training (handgrip muscles) in the brachial artery (Thijssen et al., 2009a), as well as in vessels remote to the body region involved in the exercises (i.e., improvement in brachial artery after a respiratory muscle training or single leg kick training) (Wray et al., 2006; Bisconti et al., 2018).

In a previous study Wray et al. (2006) investigated the effects of 6 weeks of a similar KE training on brachial, superficial and deep femoral arteries flow mediated dilation (FMD) in elderly people. KE training positively affected brachial artery FMD, with no changes in both superficial and deep femoral arteries (Wray et al., 2006). However, the effects of KE training in the lower limb vasculature (involved with training), as well as in the upper limb (not directly involved with training) in a young population still remain to be elucidated.

Small muscle mass exercise is being employed to maximize vascular adaptation due to its ability to circumvent central limitations and sympathetic restraint of limb 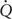 (Richardson and Saltin, 1998; Richardson et al., 1998, 2004; Esposito et al., 2018). In particular, KE exercise determines a significant change in the peripheral hemodynamics (Paterson et al., 2005) without overloading the cardiopulmonary system (Richardson et al., 2004; Esposito et al., 2010, 2011, 2018).

Compared to cycle exercise (Saito and Tsukanaka, 2019), an attenuate muscle sympathetic outflow likely occurs during KE due to a lower cardiopulmonary engagement (Esposito et al., 2010). This is so not only in healthy population (Richardson and Saltin, 1998; Richardson et al., 1998) but also in patients with central (cardiac and/or pulmonary) exercise limitation (Richardson et al., 2004; Esposito et al., 2018). In addition, this exercise modality could be more easily employed in clinical populations who have significant exercise intolerance during large muscle mass exercise due to central limitation (Esposito et al., 2011). Taking all into account, KE exercise training represents a good exercise paradigm to be used in presence of exercise intolerance due to central limitation or presence of sympatho-excitation.

On these bases, this study sought to evaluate whether small muscle mass (KE) training would induce vascular conditioning not only in the vasculature of the limb directly involved with training (as results of the femoral artery measurement) but also in vasculature of a limb not directly involved with training (as results of the brachial artery measurement). The hypothesis is that the repetitive training-induced stimuli may occur also in arteries of non-trained districts via a systemic increase in 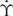.

## Materials and Methods

### Participants

Ten young, healthy participants [five females, and five males; age: 23 ± 3 years; stature: 1.70 ± 0.11 m; body mass: 66 ± 11 kg; body mass index: 23 ± 1 kg.m^-2^; mean ± standard deviation (SD)] were enrolled in the study. After full explanation of the purpose and the procedures of the study, participants signed an informed consent form. Exclusion criteria were: (i) presence of neurological, vascular and musculoskeletal impairments at the lower and upper limbs level; (ii) being on pharmacological therapy related to neural and/or vascular response, including hormonal contraceptives and oral supplements; (iii) being a smoker; (iv) systolic arterial pressure higher than 140 mmHg; and (v) having an irregular menstrual cycle (26–35 days) up to 3 months before the beginning of the study. The Institutional Review Board of the Università degli Studi di Milano approved the study, which was performed in accordance with the latest Helsinki’s Declaration principles.

### Study Design

Before testing procedures, participants underwent a preliminary session during which they familiarized with the dynamic knee extension ergometer and with the procedure to identify the maximum isometric voluntary contraction (MVC) of the knee extensor muscles of the dominant limb. During this visit, the passive limb movement (PLM) and the FMD tests (see below for a full explanation of the procedures) were performed on each participant. At the end of the tests, the ultrasound probe position for testing was marked on a transparency sheet, together with some skin landmarks (moles, scars, angiomas, etc.) for reliability purposes. The PLM and FMD outcomes obtained during the familiarization and the pre-training experimental session were used to calculate intersession reliability.

Pre- and post-8 weeks of KE training, participants were tested at the same time of the day in a climate-controlled laboratory (constant temperature of 20 ± 1°C and relative humidity of 50 ± 5%) to minimize any possible confounds due to circadian rhythm. For females, the tests were assessed on the same day of the menstrual cycle (early follicular phase days 3 ± 3). Female participants recorded their menstrual cycle in a personal diary throughout the study, which was used to assess the early follicular phase and allowed the subjects to be tested on the same menstrual day pre- and -post KE training. On the test days, participants came to the laboratory after fasting overnight, abstaining from caffeine and other similar substances for at least 12 h, and not participating in heavy exercise for at least 48 h prior the tests. Post-training assessments were performed 48 h after the last KE training session. This period was observed to prevent possible biases in measurements introduced by the acute effects of the last training session. During the first session of KE training, the possible increase in brachial artery 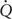 (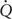
_brac_) was also determined.

### Measurements and Data Analysis

#### Maximum Work Rate

**FIGURE 1:**
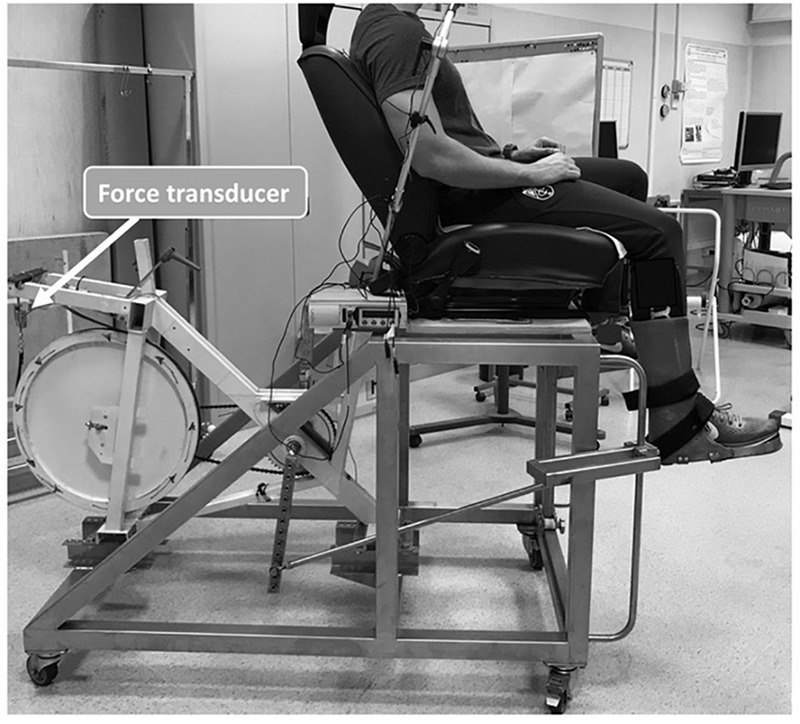
Photographic representation of participant’s positioning on the knee extension ergometer. The load-cell to calculate mechanical power is indicated by the arrow.

The maximum work rate (MWR) was determined by an incremental square wave test on a dynamic knee extension ergometer (Andersen et al., 1985). As shown in Figure 1, the test was performed while sitting on an adjustable chair in order to fit body sizes of different dimensions. Both knees were flexed at 90°, with the ankle of the dominant limb connected to a bicycle ergometer pedal arm by a rigid bar. The concentric phase occurred actively from 90° of the knee to full extension, while the eccentric phase was completely passive, driven by the flywheel momentum. The mechanical brake applied to the ergometer and the pedal frequency were measured to determine the mechanical power output. The mechanical friction, i.e., the force applied to each revolution, was measured by a force transducer (mod. SM-100 N, Interface, Crowthorne, United Kingdom), while the pedal frequency was determined by a magnetic transducer integrated in the cycle ergometer (mod. 839E, Monark, Vansbro, Sweden). Both the force and the pedal frequency signals were amplified (gain × 100) and acquired by a personal computer after an analog-to-digital conversion (model UM150, Biopac System, CA, United States) at a sampling rate of 1 kHz.

The square wave test started with an initial work load of 20 W for males and 10 W for females. Loads increased by similar amplitude steps (+20 W for male and +10 W for female) until exhaustion. Each load was maintained for 3 min, with 5 min recovery in between. The load of the last completed step was considered as MWR. The amplitude of the load increments during the square wave test was chosen as the best compromise between the identification of the maximum workload and the necessity to minimize the onset of muscle fatigue. A non-invasive impedance cardiograph device (Physio Flow^®^, Manatec Biomedical, Paris, France) was used to assess the cardiac output (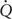
_T_), stroke volume (*q*) and heart rate (*f*_H_). At rest and during test, pulmonary 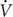
O_2_ was detected on a breath-by-breath modality by gas analysers (mod. Quark b^2^, Cosmed, Rome, Italy). The system was calibrated before each test with gas mixtures of known concentrations (16% O_2_; 5% CO_2_; balance N_2_). Data were averaged on the last 60 s of baseline and the last 30 s of the last work load. The average 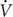
O_2_ matched to MWR was considered as peak 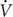
O_2_ (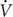
O_2peak_).

#### Maximum Isometric Voluntary Contraction

Knee extensors MVC was measured with the participants sitting on an ergometer with the hip and the knee flexed at 90° and firmly secured at the ankle level by a Velcro^®^ strap (Velcro Industries Inc., Willemstad, Netherlands Antilles) to a load cell (mod. SM-2000N operating linearly between 0 and 2000 N; Interface, Crowthorne, United Kingdom) for the force signal detection. After a standardized warm-up (10 × 2-s contractions at 50% MVC previously determined during familiarization), three MVC attempts were performed. The participants were instructed to push as fast and hard as possible for 3 s. Each MVC attempt was interspersed by 3 min of recovery. The force signal was driven to an A/D converter (mod. UM 150, Biopac, Biopac System Inc., Santa Barbara, CA, United States), sampled at 1000 Hz, and stored on a personal computer. The maximum force value recorded during tests was considered as MVC and was inserted into the data analysis.

#### Knee Extensor Muscles Volume

With thigh length, circumferences, and skinfold measurements, knee extensor muscles volume was calculated to allow an estimate of quadriceps muscle mass, as previously published in other studies (Jones and Pearson, 1969; Andersen et al., 1985; Esposito et al., 2011, 2018).

#### Single Passive Limb Movement

Single passive limb movement was performed in accordance to the recommended procedures (Gifford and Richardson, 2017; Venturelli et al., 2017b). Sitting on a chair, subjects rested in the upright-seated position for 10 min before starting the data collection, and remained in this position until the end of the test. The PLM protocol consisted of 60 s of baseline peripheral hemodynamic data collection, followed by a single passive knee flexion and extension of 1 s, after which the leg was maintained fully extended for the remaining 59 s for the post-movement data collection. PLM was performed by a member of the research team, who moved the subject’s lower leg through a 90° range of motion at 1 Hz. Throughout the test, measurements of arterial blood velocity and vessel diameter were performed in the common femoral artery of the passively moved leg, distal to the inguinal ligament and proximal to the deep and superficial femoral bifurcation by Doppler ultrasound (mod. Logiq-7, General Electric Medical Systems, Milwaukee, WI, United States). After being positioned to an insonation angle of 60° or less, a 9-MHz linear array transducer was used to measure the mean blood velocity. The sample volume was centered and size-maximized according to vessel’s diameter (Trinity et al., 2012). Femoral artery 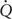 (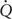
_fem_) was calculated by using data of arterial diameter and mean blood velocity as:

$$\dot{Q}(\mathrm{ml}\cdot \min^{-1})=\mathrm{mean}\;\mathrm{blood}\;\mathrm{velocity}\cdot \pi \cdot {\left(\mathrm{vessel}\;\mathrm{diameter}/2\right)}^{2}\cdot 60$$

#### Flow Mediated Dilation

Flow mediated dilation measurements were performed according to recommended procedures (Harris et al., 2010; Bisconti et al., 2018). Before FMD, the subjects laid supine for approximately 20 min to restore baseline values of cardiovascular parameters. An arterial pressure cuff was placed on the forearm immediately distal to the olecranon process in order to provide an ischemic stimulus on the forearm when inflated. Following baseline assessments, the forearm blood pressure cuff was inflated to 250 mmHg for 5 min. Brachial diameter and flow velocity recordings resumed at baseline, 30 s prior to cuff deflation and continued for 2 min post-deflation, in accordance with previous literature (Corretti et al., 2002; Harris et al., 2010; Wray et al., 2013). A 9-MHz linear array transducer attached to a high-resolution ultrasound machine was used to image the brachial artery in the distal third of the upper arm above the cuff placement. When an optimal image was obtained, the probe was held stable and longitudinal in B-mode, acquiring images of the lumen-arterial wall interface. Continuous Doppler velocity assessments were also obtained and collected using the lowest possible insonation angle (<60°). The Doppler ultrasound settings were maintained consistent pre vs. post-KE assessment among subjects. Additionally, anatomical marks were considered to ensure the same ultrasound probe, as well as pressure cuff position between the two visits. The FMD data were exported in AVI format and analyzed using commercially available software (Brachial Artery Analyzer for Research, Medical Imaging Applications, LLC, Coralville, IA, United States), in which the lumen diameter, distance between upper intima-media to lower intima media, was measured from within the same region-of-interest (Faita et al., 2011; Ratcliffe et al., 2017). This method is largely independent of investigator bias (Faita et al., 2011; Ratcliffe et al., 2017). FMD was quantified as the maximal change in brachial artery diameter after cuff release, expressed as a percentage increase (%Δ) above baseline:

(Maximum− rest diameter)/rest diameter×100

Brachial artery 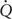 (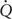
_brac_) was calculated as previously described for PLM assessments.


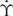
 was calculated post-cuff release using the following equation:

$$\dot{ϒ}\;(s^{-1})\;=\;8\;v_{\mathrm{mean}}/\mathrm{vessel}\;\mathrm{diameter}$$

The cumulative 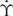, corresponding to the reactive hyperaemia post-cuff release (total 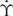 from cuff release to time-to-peak), was integrated (AUC) by using the trapezoidal rule, and calculated as:

(4) $$\sum [y_{i}\cdot (x_{i+1}{-x}_{i})+\frac{1}{2}(y_{i+1}{-y}_{i})\cdot (x_{i+1}{-x}_{i})]$$

The 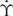 AUC reflects the amount of mechanical stimulus applied on the endothelium during the cuff release hyperaemic response until time-to-peak. Considering that FMD is primarily dependent on the endothelium response to mechanical stimuli, the FMD was therefore divided by cumulative 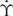 (FMD/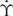) (Pyke and Tschakovsky, 2005).

### Isolated Knee Extension Muscles Training

**FIGURE 2:**
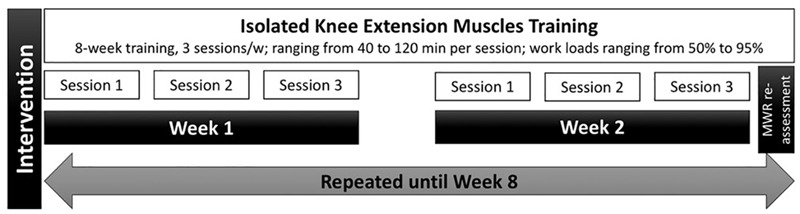
Schematic chart of the experimental design for isolated knee extension muscles training.

A schematic drawn of the KE training design is given in Figure 2.

Similar to previous studies (Esposito et al., 2011, 2018), participants underwent an 8-week KE training (3 sessions.w^-1^) involving both legs, one at a time. The KE training was performed on the same ergometer used for MWR assessment, which allows participants to train only a single limb leg extensor muscles. The KE training was similar to that proposed in a previous study (Esposito et al., 2010). Briefly, workloads ranged mainly from 50% to 95% MWR, with an average session duration of 40 min for each leg (a report of the characteristics of each KE training session is provided as Supplementary Table S1). The session’s workloads were readjusted every 2 weeks reassessing a new MWR. Each training session was supervised by an expert operator, who monitored the attendance, the correct exercise execution, and the maintenance of the workload. The participants not attending at least the 80% of the total training sessions were excluded from the study. In this case, a new participant was recruited to replace the drop out.

To assess the acute effects of exercise on 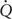
_fem_ and 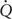
_brac_, the related 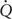, together with 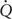
_T_, *q, f*_H_, and mean arterial pressure (MAP) were measured during the first 6 min of the first session of KE training (performed at 50% of MWR, representing the minimum workload intensity within the training). Femoral and brachial artery mean blood velocity and diameter were assessed by ultrasound. As previously described (Venturelli et al., 2017a), a modelflow method (Finapres Medical System) automatically assessed the *q*, with 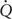
_T_ calculated as the product of *q* and *f*_H_. *f*_H_ and the MAP were detected by electrocardiography and finger photoplethysmography positioned at the heart level (Finometer PRO, Finapres Medical System, Amsterdam, Netherlands). Before starting the bout of exercise training, a baseline measurement of 30 s was recorded. From mean blood velocity and diameter assessments during the last minute of exercise, the 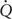
_fem_, 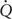
_brac_ and the 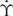 at femoral (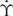
_fem_) and brachial artery (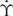
_brac_) level were calculated, together with the central hemodynamic parameters.

### Statistical Analysis

Statistical analysis was performed using a statistical software package (IBM SPSS Statistics v. 22, Armonk, NY, United States). Shapiro–Wilk test was used to check the normal distribution of the sampling. Based on a previous work in which percentage changes of about 45% were detected in brachial artery FMD/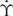 (main outcome) after a training protocol not directly involving the upper limbs (Bisconti et al., 2018), a sample size of ten participants was selected to ensure a statistical power > 0.80 and a type-1 error < 0.05, To determine intersession reliability in the endothelial function parameters, the intraclass correlation coefficient (ICC) and percentage change of the standard error of the measurement (SEM%) were calculated. The ICC was interpreted as follows: >0.90: very high; 0.89–0.70: high; 0.69–0.50: moderate (Munro, 2004). The minimal detectable change at 95% confidence interval (MDC_95%_) was used to detect sensitivity of the effects on endothelial function parameters between pre- and post-isolated knee extension muscles training (Donoghue et al., 2009). To assess significant effects of KE training on MWR, PLM and FMD parameters, a paired Student’s *t*-test was applied pre and post data. Statistical significance was positioned at *p* < 0.05. Effect sizes measure expressed as Cohen’s *d* was calculated for each parameter to quantify within-group magnitude changes (Cohen, 1992). Cohen’s *d* value was classified as trivial for values < 0.19, small between 0.2 and 0.6, moderate between 0.6 and 1.2, large between 1.2 and 2.0, and very large > 2.0. If not otherwise stated, data are presented as mean ± standard deviation.

## Results

Participants’ attendance was about 95% (228/240 training sessions). Two participants dropped out throughout the study because of injury (not related to the training protocol) and lack of time. They were immediately replaced in order to maintain the required sample size.

### Reliability

Intersession reliability for endothelial function parameters is reported in Table 1. ICC and SEM% in PLM parameters ranged from 0.997 and 0.37 to 0.999 and 2.95, respectively. ICC and SEM% in FMD parameters spanned from 0.701 and 1.47 to 0.997 and 8.98, respectively. In both PLM and FMD, MDC_95%_ was comprised between 1 and 25%.

**Table 1:** Intersession reliability (intraclass correlation coefficient, ICC; standard error of measurement as a percentage, SEM%), and sensitivity (minimum detectable change at 95% confidence interval, MDC_95%_) values in the main endothelial function parameters calculated during the single passive limb movement (PLM) and flow mediated dilation (FMD) tests.

		Trial 1	Trial 2	ICC	SEM%	MDC_95%_
PLM	Femoral artery diameter (cm)	0.78 ± 0.09	0.78 ± 0.09	0.999	0.37	1.01
	Rest 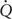 _fem_ (ml ⋅ min^-1^)	224 ± 116	228 ± 113	0.998	2.27	6.28
	Max 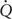 _fem_ (ml ⋅ min^-1^)	552 ± 305	538 ± 282	0.997	2.95	8.18
	AUC (ml)	3042 ± 2392	3078 ± 2370	0.999	2.46	6.82
FMD	Rest brachial artery diameter (cm)	0.29 ± 0.07	0.28 ± 0.08	0.997	1.47	4.07
	Peak brachial artery diameter (cm)	0.36 ± 0.08	0.38 ± 0.08	0.936	5.17	14.32
	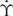 AUC (s^-1^; × 1000)	292 ± 39	294 ± 42	0.701	8.98	25.42

*Femoral blood flow at rest and at maximum value after PLM maneuvre, 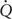
_*fem*_; shear rate, 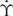; area under the curve of the 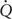 and 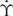 as a function of time in PLM and FMD, respectively, AUC. Data are presented as mean ± SD.*

### Acute 50% MWR Exercise

Central hemodynamic and peripheral blood parameters assessed during the first 6 min of the first session of KE training (50% MWR) are presented in Table 2. All the parameters were significantly higher at the end of the sixth minute of exercise compared to baseline. In details, 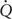
_T_, *q, f*_H_ and MAP increased by 114, 16, 85, and 18%, respectively. Both 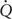
_fem_ and 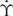
_fem_ increased by about fivefolds, whereas 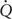
_brac_ and 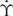
_brac_ increased by 26 and 60%, respectively.

**Table 2:** Central hemodynamic parameters, femoral, and brachial artery blood flow detected at baseline and at the end of the sixth minute during the first session of isolated knee extension muscles training performed at 50% of the maximum work rate.

	Baseline	Sixth minute	Paired Student’s *t*-test	Cohen’s *d*
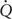 _T_ (l ⋅ min^-1^)	6.43 ± 0.48	13.75 ± 1.02	*t*_(9)_: -20.53; *p* < 0.001	-8.79
*q* (ml)	95 ± 9	110 ± 10	*t*_(9)_: -3.48; *p =* 0.003	-1.49
*f*_H_ (bpm)	68 ± 7	125 ± 13	*t*_(9)_: -12.21; *p* < 0.001	-5.23
MAP (mmHg)	99 ± 9	117 ± 11	*t*_(9)_: -4.17; *p* < 0.001	-1.78
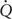 _fem_ (ml ⋅ min^-1^)	284 ± 150	1759 ± 646	*t*_(9)_: -7.04; *p* < 0.001	-2.87
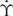 _fem_ (s^-1^)	11.61 ± 6.13	72.04 ± 26.45	*t*_(9)_: -7.35; *p* < 0.001	-3.01
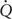 _brac_ (ml ⋅ min^-1^)	34 ± 3	43 ± 5	*t*_(9)_: -4.31; *p* < 0.001	-1.85
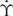 _brac_ (s^-1^)	14.80 ± 3.92	23.70 ± 8.14	*t*_(9)_: -3.13; *p =* 0.006	-1.34

*Cardiac output, 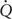
_*T*_; stroke volume, *q*; heart rate, *f*_*H*_; mean arterial pressure, MAP; femoral artery blood flow, 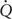
_*fem*_; femoral artery shear rate, 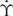
_*fem*_; brachial artery blood flow, 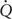
_*brac*_; brachial artery shear rate, 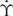
_*brac*_. Data are presented as mean ± SD.*

### Isolated Knee Extension Muscles Training

As shown in Table 3, MWR, MVC, KE muscle volume, and 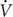
O_2peak_ increased significantly by 44, 21, 7 and 11%, respectively, after KE training. No significant changes were retrieved in the other central hemodynamic parameters, both at rest and at MWR.

**Table 3:** Maximum knee-extension muscles (KE), isometric voluntary contraction (MVC), work rate (MWR), KE volume, and central hemodynamic parameters before (Pre) and after (Post) KE training.

	Pre	Post	Paired Student’s *t*-test	Cohen’s *d*
MWR (W)	48 ± 13	69 ± 21	*t*_(9)_: -2.69; *p =* 0.015	-1.15
MVC (N)	501 ± 17	604 ± 23	*t*_(9)_: -11.39; *p <* 0.001	-4.88
KE volume (cm^3^)	3919 ± 355	4199 ± 452	*t*_(9)_: -2.17; *p =* 0.003	-1.69
Rest 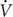 O_2_ (ml ⋅ kg^-1^ ⋅ min^-1^)	4.0 ± 0.8	4.1 ± 0.7	*t*_(9)_: 0.20; *p =* 0.844	-0.09
Peak 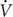 O_2_ (ml ⋅ kg^-1^ ⋅ min^-1^)	22.7 ± 3.8	25.1 ± 4.2	*t*_(9)_: -1.98; *p =* 0.042	-0.94
Rest 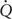 _T_ (l ⋅ min^-1^)	5.47 ± 2.26	6.19 ± 1.35	*t*_(9)_: -0.87; *p =* 0.398	-0.37
Peak 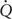 _T_ (l ⋅ min^-1^)	15.11 ± 7.24	15.68 ± 4.04	*t*_(9)_: -0.22; *p =* 0.830	-0.09
Rest *q* (ml)	77.05 ± 37.00	83.57 ± 22.18	*t*_(9)_: -0.48; *p =* 0.638	-0.20
Peak *q* (ml)	100.39 ± 43.47	106.29 ± 27.63	*t*_(9)_: -0.37; *p =* 0.360	-0.16
Rest *f*_H_ (bpm)	72 ± 8	72 ± 8	*t*_(9)_: 0.00; *p =* 1.000	0.00
Peak *f*_H_ (bpm)	148 ± 22	147 ± 19	*t*_(9)_: 0.11; *p =* 0.915	0.05

*Pulmonary oxygen uptake, 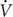
O_*2*_; cardiac output, 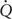
_*T*_; stroke volume, *q*; heart rate*: f*_*H*_. Data are presented as mean ± SD.*

### Single Passive Limb Movement and Flow Mediated Dilation


**FIGURE 3:**
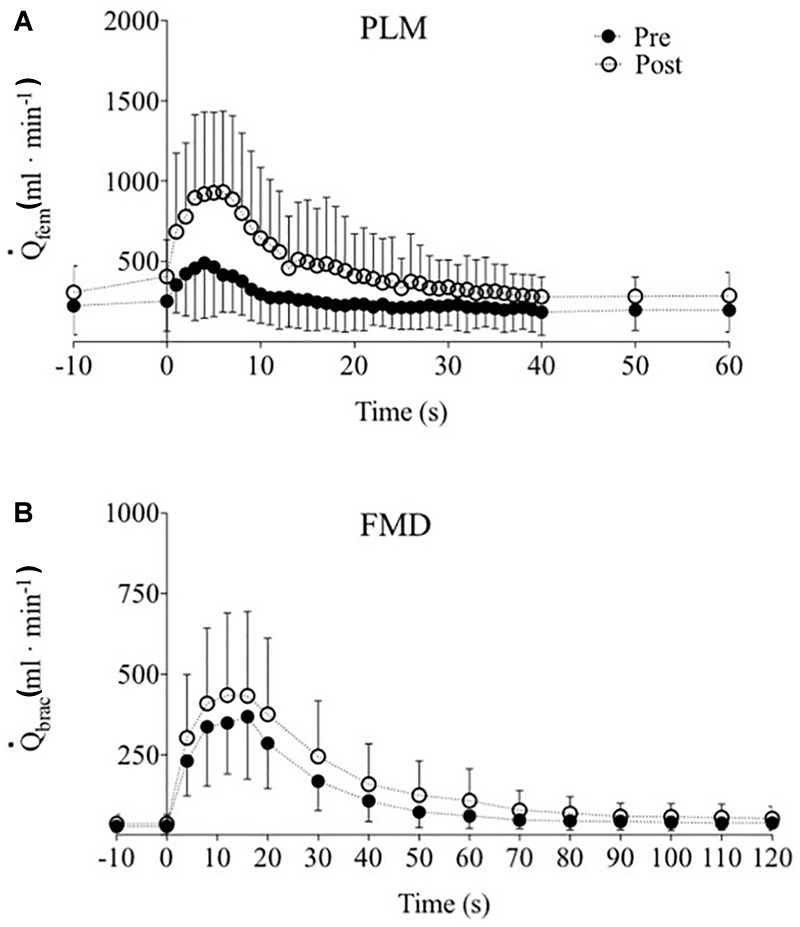
Femoral and brachial blood flow (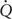
_fem_ and 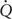
_brac_, respectively) hyperaemic response to single passive limb movement (PLM, **A**) and flow mediated dilatation (FMD, **B**) tests pre- (closed circles) and post- (open circles) training. Data are presented as mean ± SD.

**FIGURE 4:**
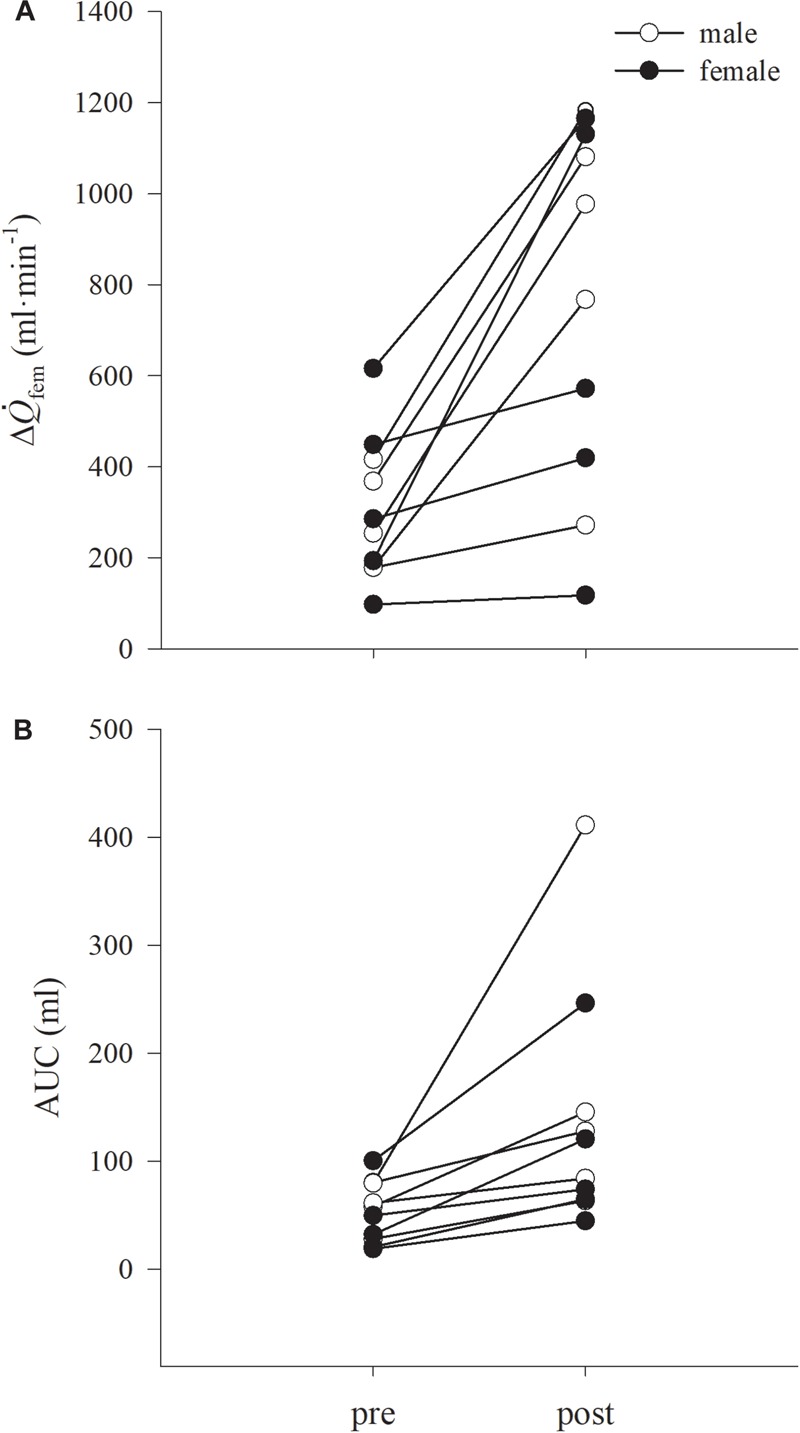
Individual data for changes in difference between rest and maximum femoral artery blood flow during passive limb movement (PLM), (Δ
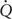
_fem_, **A**) and cumulative blood flow, expressed as AUC **(B)**, are shown. Males and females are represented in open and closed circles, respectively. Data are presented as mean ± SD.

**FIGURE 5:**
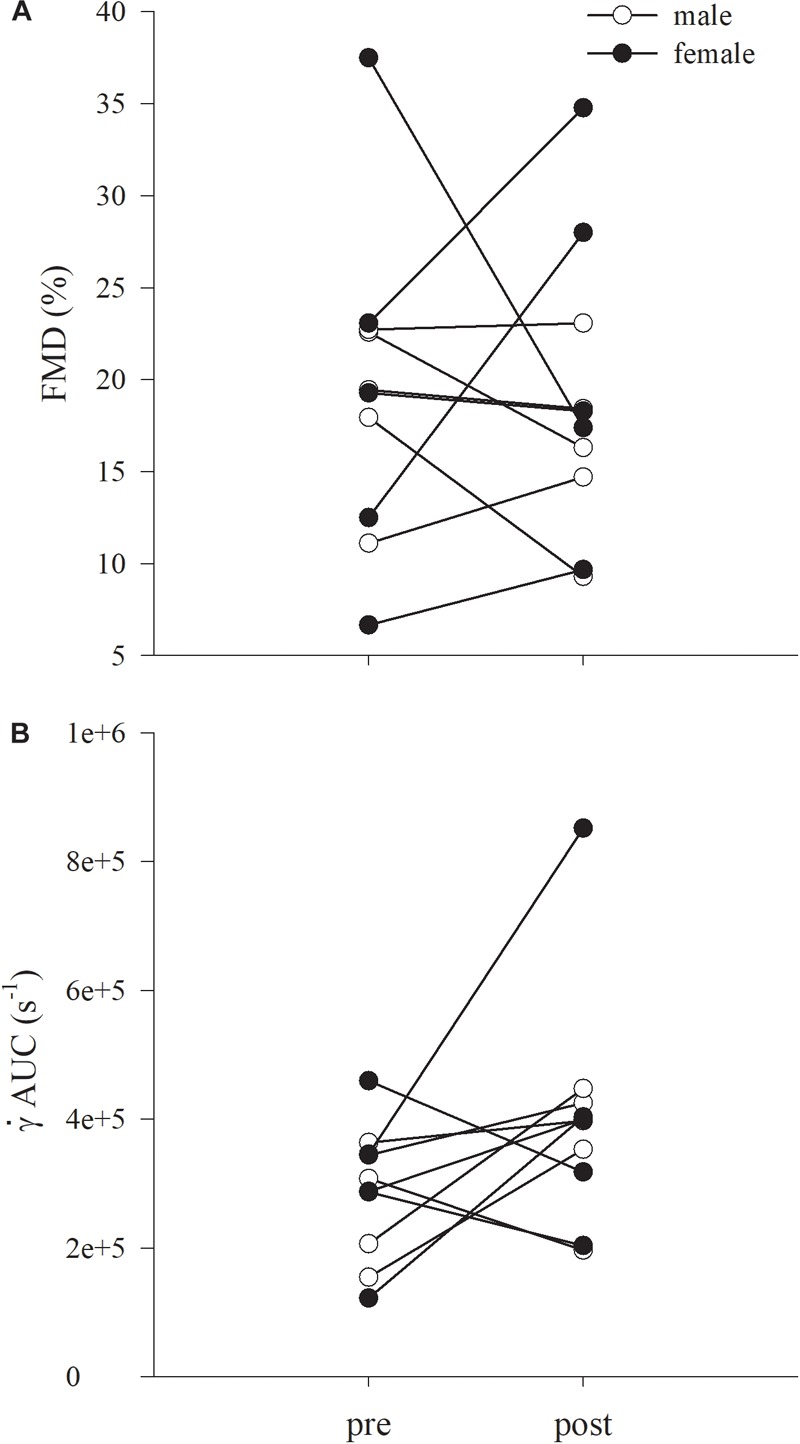
Individual data for brachial artery flow mediated dilatation (FMD) **(A)** and cumulative shear expressed as AUC (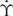 AUC **B**), are shown. Males and females are represented in open and closed circles, respectively. Data are presented as mean ± SD.

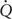
_fem_ kinetics response to PLM is illustrated in Figure 3A. 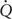
_fem_ AUC, maximum 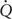
_fem_, (max 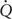
_fem_), and Δ
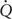
_fem_ (maximum - resting 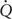
_fem_) are reported in Table 4. The pre-post changes in Δ
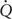
_fem_ and 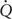
_fem_ AUC for each participant are presented in Figure 4. After training, 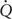
_fem_ AUC, max 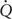
_fem_ and Δ
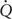
_fem_ increased significantly by 161, 104, and 153 compared to pre-training, respectively (Table 4 and Figure 3A). The pre-post changes in FMD and 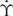 AUC for each participant are provided in Figure 5. No significant differences were detected after 8 weeks of KE training in any FMD parameter (Table 4 and Figure 3B).

**Table 4:** Changes in femoral and brachial arteries endothelial function parameters before (Pre) and after (Post) isolated knee extensor muscles training.

		Pre	Post	Paired Student’s *t*-test	Cohen’s *d*
PLM	AUC (ml)	53 ± 28	138 ± 112	*t*_(9)_: -2.33; *p =* 0.032	-1.00
	Femoral artery diameter (cm)	0.77 ± 0.09	0.78 ± 0.09	*t*_(9)_: -0.25; *p* = 0.807	-0.11
	Max 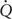 _fem_(l ⋅ min^-1^)	528 ± 318	1078 ± 505	*t*_(9)_: -2.91; *p <* 0.001	-1.25
	Δ 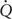 _fem_ (l ⋅ min^-1^)	304 ± 158	769 ± 399	*t*_(9)_: -3.43; *p =* 0.003	-1.47
FMD	FMD (%)	19 ± 9	18 ± 9	*t*_(9)_: 0.25; *p =* 0.807	0.11
	Rest brachial artery diameter (cm)	0.29 ± 0.08	0.31 ± 0.07	*t*_(9)_: -0.60; *p =* 0.559	-0.25
	Peak diameter (cm)	0.35 ± 0.08	0.37 ± 0.08	*t*_(9)_: -0.59; *p =* 0.583	-0.24
	Time-to-peak (s)	27 ± 11	26 ± 8	*t*_(9)_: 0.23; *p =* 0.819	0.10
	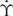 AUC (s^-1^; × 1000)	294 ± 42	399 ± 192	*t*_(9)_: -1.69; *p =* 0.108	-0.72
	FMD/ 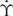 (%/s^-1^)	0.06 ± 0.03	0.05 ± 0.03	*t*_(9)_: 0.75; *p =* 0.466	0.32
	AUC (ml)	30.62 ± 14.76	40.81 ± 25.72	*t*_(9)_: -1.09; *p =* 0.292	-0.47

*Single passive limb movement, PLM; area under the curve of the blood flow and 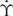 as a function of time in PLM and FMD, respectively, AUC; femoral artery maximum blood flow, max 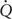
_*fem*_; difference between rest and maximum femoral artery blood flow, Δ
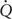
_*fem*_; flow mediated dilation, FMD; shear rate, 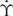. Data are presented as mean ± SD.*

## Discussion

This study sought to investigate possible positive effects of KE training on the endothelial function in artery of a limb not directly involved with training, such as the brachial artery. The main finding was that KE training increased endothelial function only in the lower limb, assessed by the femoral artery measurements, without any significant change in the upper limb, assessed by brachial artery measurements. Despite the increase during every exercise session in peripheral 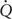 and, in turn, in 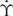 in the brachial artery (limb not directly involved with KE training), the mechanical stimulus in that area was not strong enough to trigger the chain of events turning to an endothelial function enhancement in the brachial artery.

### Cardiac, Metabolic, and Skeletal Muscle Response to KE Training

The KE training protocol led to marked improvements at the peripheral level with no changes in heart hemodynamics due to the minimum taxing of this small muscle mass exercise paradigm on central factors. These findings are in line with previous reports in health and disease (Andersen et al., 1985; Richardson et al., 2004; Esposito et al., 2010, 2011) and indicate that this specific training paradigm can induce improvements in peripheral convective and diffusive oxygen transport without detectable changes in central hemodynamic. The 44% increase in MWR is only partially supported by the enhancement in 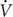
O_2peak_ (+11%). Indeed, the larger improvement in MVC (+21%) than in 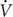
O_2peak_, accompanied by the increase in estimated leg extensor muscle volume (+7%) suggested that the higher post KE training MWR was also induced by other metabolic pathways beside the aerobic power. These findings are somewhat in agreement with a previous investigation in chronic heart failure participant engaged in an 8-week KE training at 65/75% MWR (Magnusson et al., 1996). After training, the authors demonstrated an increment of the MWR not accompanied by increases in 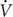
O_2peak_, whereas the knee extensor muscles MVC and mass were increased.

### Effect of KE Training on Vascular Endothelial Function

It was previously demonstrated that KE training is an effective strategy to highly challenge the peripheral skeletal muscles, involved with exercise, without taxing central heart and respiratory function (Andersen et al., 1985). This training modality is particularly useful to counteract sarcopenia in the elderly and to decrease exercise intolerance in patients with central hemodynamic limitation, such as heart failure or chronic obstructive pulmonary disease (Richardson et al., 2004; Esposito et al., 2010, 2011, 2018). After 8 weeks of KE training, the endothelial function in the femoral artery, which was directly involved with KE training, increased as shown in Table 4. Contrary to the experimental hypothesis, this was not the case in the brachial artery.

As a result of KE training, all the PLM-related parameters, such as PLM AUC, max 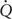
_fem_ and Δ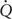, increased together with MWR, highlighting the positive effect of this type of training not only on the mechanical power (MWR) but also on the functionality of the lower limb vasculature directly exposed to exercise. Remarkably, despite PLM is a relative new research tool, its response provides an important insight into the function of the vascular system with clinical relevance. The most representative and common factors reported to describe the PLM response are: (i) the peak flow; (ii) the change from baseline to peak flow (Δ peak flow); and (iii) AUC (Gifford and Richardson, 2017). As previously reported, all these parameters are strictly related to NO bioavailability (Gifford and Richardson, 2017). Considering vascular tube length and blood viscosity relatively constant, and applying the Poiseuille’s law, PLM-induced hyperemia might be driven by two main factors: an increased perfusion pressure and an increased peripheral vasodilation (i.e., decreased vascular tone). In detail, the vascular endothelium seems to play an important role in the PLM-induced change in vascular tone (Mortensen et al., 2012; Trinity et al., 2012, 2015; Groot et al., 2015). Indeed, the passive movement of the leg causes the mechanical deformation of vessels determining also NO release (Cheng et al., 2009; Jufri et al., 2015), that, in turn, results in the dilatation of the vascular bed (Mortensen et al., 2012; Trinity et al., 2012). Therefore, considering the nature of this hyperemic response, we can ascribe the present improvement in PLM’s data to an enhanced NO-bioavailability (Trinity et al., 2015; Venturelli et al., 2017b). Oscillations in 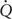 during training sessions, indeed, may be an important stimulus to the endothelial cell membrane deformation that trigger a series of signaling events favoring NO production, as previously mentioned in the introduction (Green et al., 2005).

However, it is important to underline that although PLM and FMD refer to two different part of the circulation (micro- and macro-circulation, respectively) (Eskurza et al., 2001; Mortensen et al., 2012), a relationship exists between these two different type of vascular assessments (Rossman et al., 2016), possibly due to the influence of reactive hyperemia/shear rate on FMD (Padilla et al., 2008), which is itself largely determined by the microcirculation (Pyke and Tschakovsky, 2005).

To date a consistent body of literature reported the positive effect of a small muscle training (i.e., handgrip training) on endothelial function (Tinken et al., 2010; Credeur et al., 2012; Badrov et al., 2016). Even in these studies, the positive effects of training on the vasculature were primarily ascribed to the 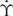 stimulus acting on the inner vessel lay and leading to an improvement in endothelial cells response (Tinken et al., 2010; Credeur et al., 2012; Badrov et al., 2016), likely due to an increase in the NO-bioavailability (Corretti et al., 2002; Harris et al., 2010; Tinken et al., 2010; Thijssen et al., 2011; Green et al., 2017a).

Our data in acute condition (Table 2) indicate that during acute KE exercise an increase in peripheral circulation occurred also at the brachial artery level. This observation confirmed our hypothesis that the 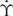 stimulus increased in limbs not directly involved with training, and possibly could have led to positive effects also in the endothelial cells response. As a matter of fact, chronic 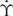 stimulus occurring in the brachial artery during different training modalities not directly involving upper limb muscles (such as cycling or respiratory muscle exercise) led to enhancements in brachial artery endothelial function (Birk et al., 2012a; Bisconti et al., 2018). More in details, acute cycle exercise increased 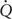
_brac_ by about 30% with respect to baseline (Green et al., 2005) and 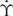
_brac_ by about +280% (Birk et al., 2012a). Respiratory muscles exercise increased 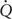
_brac_ up to +241% from baseline, with a 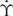
_brac_ increase by about +115%. In the present study, KE exercise increased 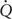
_fem_ by about +519% from baseline, while 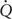
_brac_ increased only by +26%, generating a different level of mechanical stress (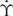
_fem_ and 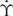
_brac_ +520 and +60%, respectively). Given that the positive effects of 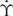 on the endothelial function are likely dependent on the magnitude with which the 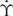 stimulus acts on the vessel (Green et al., 2011), the difference in 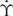 in the two districts during exercise may explain the lack of training effects on brachial artery endothelial properties. A major strength of this study is the PLM data following KE training. As mentioned before, this significant finding of improved vascular function following 8 weeks of training contradicts what is reported with the FMD model and may implicate PLM as a more robust measure of vascular adaptation/health in response to increased exercise/physical activity patterns, thus explaining some discrepancies with previous reports (Wray et al., 2006).

However, it should be also taken into account that at the end of training the increase in the PLM response due to 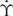 repeated stimuli does not involve the vasodilator capacity of the common femoral artery (Gifford and Richardson, 2017; Venturelli et al., 2017b), which is the largest conduit artery in the thigh with the main role of 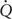 delivery rather than regulation. Therefore, another possible concomitant explanation, for the lack of effects on the brachial artery, could be that an exercise-induced 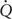 increase in the common femoral artery (with minimum vasodilator capacity) leads to a much higher 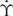 than in the brachial artery, where increases in 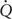 are accompanied by vasodilation.

Interestingly Wray et al. (2006) investigated the effects of 6 weeks of a similar KE training on brachial, deep and superficial femoral arteries with age. KE training positively affected brachial artery FMD, with no changes in both deep and femoral arteries (Wray et al., 2006). Such results suggest that the pre-training vascular functionality level could play an important role in determining or not some positive results. It is therefore likely that, in the face of a “normal vascular functionality”, arteries not directly involved with exercise possibly require a greater stimulus to achieve a significant improvement. An alternative mechanism to explain the lack of increase in FMD in the brachial artery after KE training could be the occurrence of a structural arterial remodeling. Studies in humans reported that during a training protocol, changes in FMD occur during the first few weeks of training before returning to pre-training values, often not accompanied by modifications of the baseline diameter (Tinken et al., 2008; Weber et al., 2013), suggesting that structural remodeling of the vessels may have likely occurred. Based on an animal model, it appears that structural arterial remodeling may occur to counteract the endothelial cells response to reactive hyperemia (i.e., %FMD) (Green et al., 2017a). Despite the model utilized (animal vs. human), adaptations in terms of vascular remodeling after a training protocol are shear-stress dependent (Tinken et al., 2008; Green et al., 2017a). Indeed, exercise training may affect vascular tissue not only by modifying the function (e.g., %FMD), but also by inducing structural modifications in baseline and peak diameter (Green et al., 2017a). In the present study, neither progressive measurements during training (e.g., after 4/6 weeks of training), nor specific tests to observed these possible structural changes [e.g., ischemic handgrip exercise (Naylor, 2005)] were performed, thus a possible vascular remodeling could have not been disclosed.

### Study Limitations

This study comes with some known limitations. First, although a sample size of ten participants was higher enough to reach a statistical power > 0.80, the enrolment of a higher number of sex-balanced participants may allow to highlight possible gender differences. Nevertheless, the individual changes in FMD% and 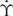 AUC did not demonstrate sex differences. Further studies are therefore needed to evaluate specific sex-related difference in brachial artery vascular response after training. Second, this study was not matched against a group of elderly and/or people presenting cardiovascular dysfunction. As mentioned in the discussion, a similar KE training model was previously demonstrated to have a positive effect on brachial artery FMD in a group of elderly without any changes in young people (Wray et al., 2006), leading to the hypothesis that the vascular health level pre-exercise training might be a pivotal factor to consider when changes in arteries functionality are expected.

Third, as previously stated, the lack of progressive FMD measurements throughout the 8-week training period could have disguised a possible brachial artery structural remodeling. However, no changes in baseline and peak brachial diameter were found after training. Moreover, in the light of the high reliability level obtained here, the lack of changes in FMD data after KE training could likely not be ascribed to the operator’s skills level. Future studies investigating the effects of KE training are necessary to reveal a possible arterial remodeling in a distal artery from exercise.

## Conclusion

The hypothesis that KE exercise could represents a paradigm able to increase peripheral 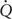 was confirmed by the increase in 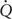
_brac_ and 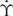
_brac_ assessed during an acute KE training session, suggesting that possible positive results could have been found also in the upper limb as results of brachial artery measurement. In the present study, though, the magnitude of this stimulus was not sufficient to promote a significant vascular conditioning in the upper limb (i.e., brachial artery FMD), as in the lower limb (i.e., femoral artery PLM). Future studies are needed to assess possible effects of KE training on arteries in districts not directly involved with training in populations with reduced endothelial function, such as patients with heart failure of chronic obstructive pulmonary disease.

## Ethics Statement

This study was carried out in accordance with the recommendations of the “Institutional Review Board of the Università degli Studi di Milano with written informed consent from all subjects. All subjects gave written informed consent in accordance with the Declaration of Helsinki. The protocol was approved by the Institutional Review Board of the Università degli Studi di Milano”.

## Author Contributions

AVB, EC, SL, MV, RG, GC, SS, SR, EL, and FE conceived and designed the study. AVB, EC, SL, MV, RG, and SS performed the experiments. AVB, EC, and SL analyzed the data. AVB, EC, SL, and FE interpreted the results. AVB and EC prepared the figures. AVB, EC, SL, and FE drafted the manuscript. AVB, EC, SL, MV, GC, SS, SR, EL, RG, and FE edited and approved the final manuscript.

## Conflict of Interest Statement

The authors declare that the research was conducted in the absence of any commercial or financial relationships that could be construed as a potential conflict of interest.

## Abbreviations

AUC: area under the curve
*f*_H_: hearth rate
FMD: flow mediated dilation
KE: isolated knee extension muscles
MAP: mean arterial pressure
MWR: maximum work rate
NO: nitric oxide
PLM: single passive limb movement
*q*: stroke volume
blood flow
_brac_: brachial blood flow
_fem_: femoral blood flow
_T_: cardiac output
O_2_: oxygen uptake
O_2peak_: peak oxygen uptake
shear rate
_brac_: brachial shear rate
_fem_: femoral shear rate
