# Supplementary material for: Evidence of Improved Vascular Function in the Arteries of Trained but Not Untrained Limbs After Isolated Knee-Extension Training
Source: Front Physiol. 2019 Jun 12;10:727. doi: 10.3389/fphys.2019.00727 (PMC6581732; doi:10.3389/fphys.2019.00727)
Supplement: Supplementary file 1 [file Table_1.DOCX]

| Isolated knee extension training | |
| --- | --- |
| Session | Work rate and duration |
| Session 1 | 25 min 50% MWR;  5 min rest;  5 min 70% MWR. |
| Session 2 | 30 min 50% MWR  5 min rest;  5 min 70% MWR. |
| Session 3 | 20 min 50% MWR  3 min rest;  5 min 70% MWR.  3 min rest;  5 min 70% MWR. |
| Session 4 | 20 min 50% MWR  3 min rest;  5 min 70% MWR.  3 min rest;  20 min 50% MWR. |
| Session 5 | 10 min 50% MWR  3 min rest;  5 min 70% MWR.  3 min rest;  5 min 70% MWR.  3 min rest;  5 min 70% MWR. |
| Session 6 | New MWR determination  30 min 50% MWR |
| Session 7 | 5 min 50% MWR  2 min rest;  6 min x 3 80% MWR with a 3-min rest in between. |
| Session 8 | 50 min 50% MWR |
| Session 9 | 5 min 50% and 5 mins 60% MWR alternated for 45 mins |
| Session 10 | 5 min 50% MWR  2 min rest;  8 min x 2 90% MWR with a 2-min rest in between;  5 min 50% MWR |
| Session 11 | 10 min 60% MWR;  2 min 30% MWR;  2 min 60% MWR;  2 min 95% MWR;  2 min 30% MWR;  2 min 60% MWR;  2 min 95% MWR;  10 min 60% MWR; |
| Session 12 | New MWR determination  30 min 50% MWR |
